# Supplementary material for: Molecular profiling of tissue biopsies reveals unique signatures associated with streptococcal necrotizing soft tissue infections
Source: Nat Commun. 2019 Aug 26;10:3846. doi: 10.1038/s41467-019-11722-8 (PMC6710258; doi:10.1038/s41467-019-11722-8)
Supplement: Supplementary file 1 — Supplementary Information [file 41467_2019_11722_MOESM1_ESM.pdf]

Supplementary Information for

Molecular profiling of tissue biopsies reveals unique signatures associated with streptococcal necrotizing soft tissue infections

Robert Thänert[1]†, Andreas Itzek[1]†, Jörn Hoßmann[1], Domenica Hamisch[1], Martin Bruun Madsen[2], Ole Hyldegaard[3], Steinar Skrede[4,5], Trond Bruun[4], Anna Norrby-Teglund[6], INFECT study group, Eva Medina[7]† & Dietmar H. Pieper[1]†[*]

[1]Microbial Interactions and Processes Research Group, Helmholtz Center for Infection Research, Braunschweig, Germany.

[3]Department of Intensive Care, Copenhagen University Hospital, Rigshospitalet, Copenhagen, Denmark.

[3]Department of Anaesthesia, Centre of Head and Orthopaedics, Copenhagen University Hospital, Rigshospitalet, Copenhagen, Denmark.

[4]Department of Medicine, Haukeland University Hospital, Bergen, Norway.

[5]Department of Clinical Science, University of Bergen, Bergen, Norway.

[6]Center for Infectious Medicine, Karolinska Institutet, Karolinska University Hospital, Huddinge, Sweden.

[7]Infection Immunity Research Group, Helmholtz Center for Infection Research, Braunschweig, Germany.

†These authors contributed equally to this work.

Supplementary Figures

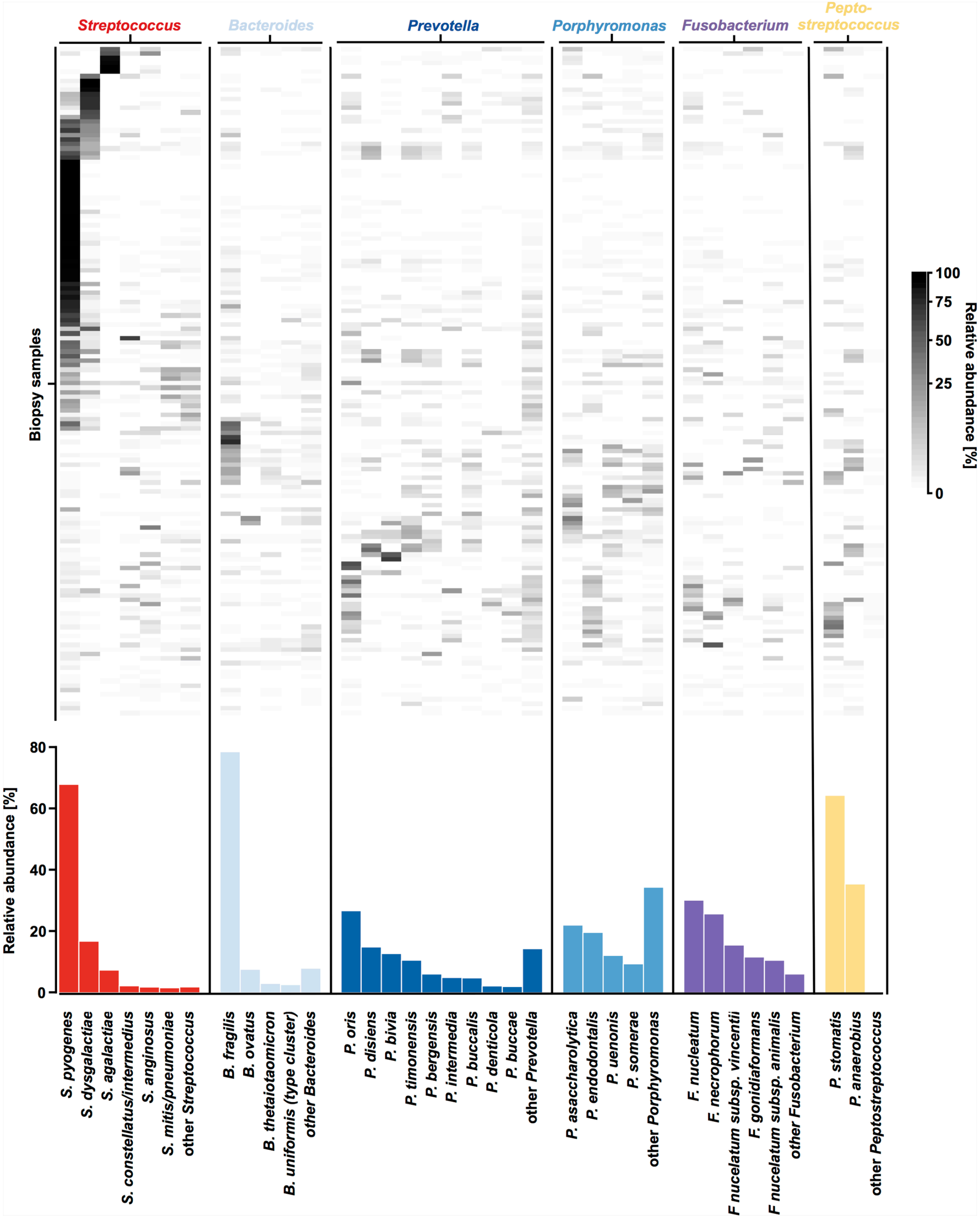

Supplementary Fig. 1: Contributional species level diversity varies between the major bacterial genera associated with NSTIs. Selected bacterial species identified in

tissue biopsies from NSTI patients. Bar charts depict the relative contribution of a respective species to the total genus abundance in the investigated cohort. Source data are provided in Supplementary Data 1.

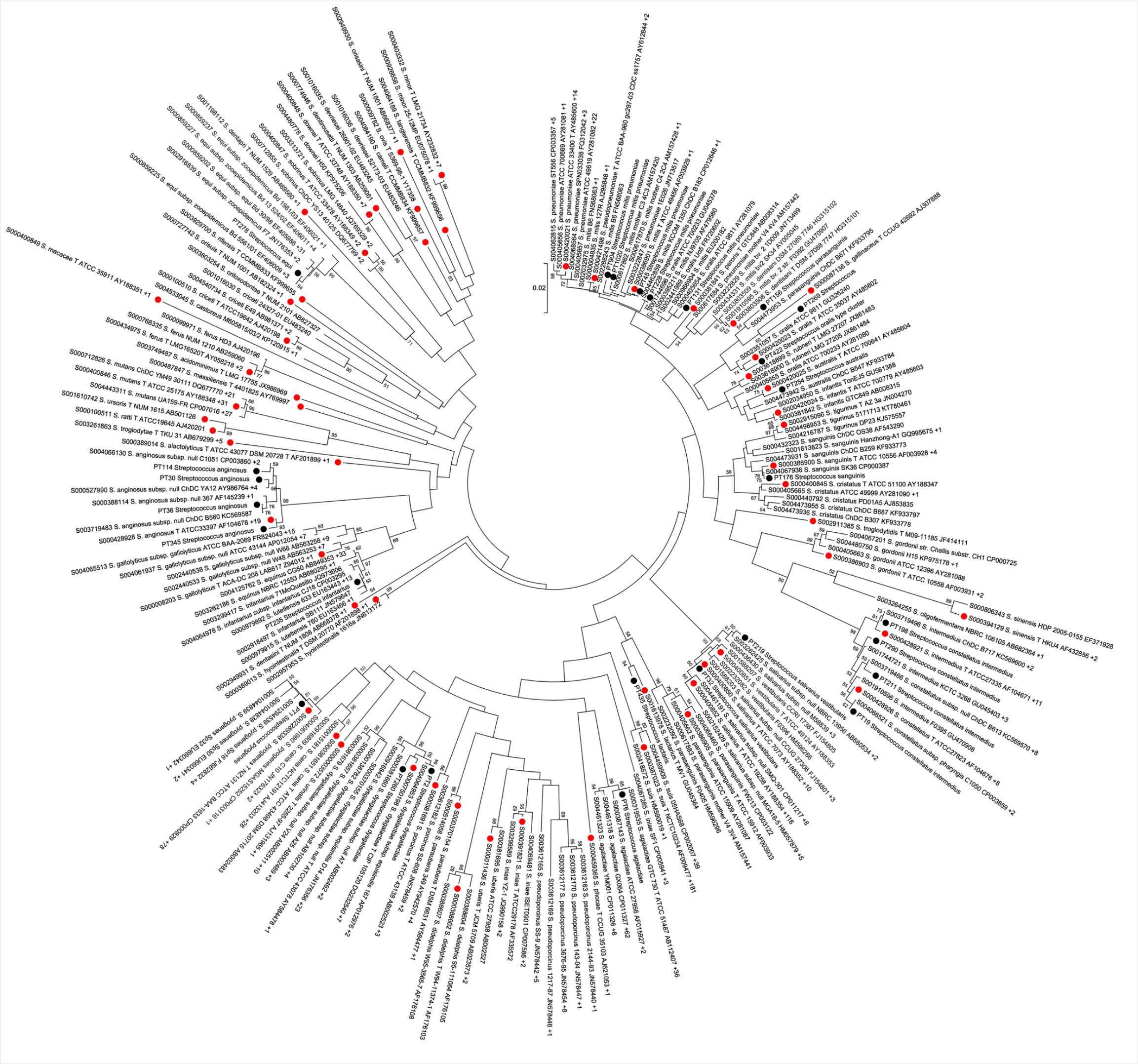

Supplementary Fig. 2: Taxonomic distribution of 16S rRNA gene sequences originating from *Streptococcus* spp. Sequences were aligned using MUSCLE and edited with SEAVIEW. The phylogenetic tree was constructed with MEGA7 using the neighbor-joining algorithm with p-distance correction and pairwise deletion of gaps and missing data. A total of 100 bootstrap replications were done to test for branch robustness (bootstrap values >50% are shown adjacent to each cluster node). Sequences of all of isolates with species level annotation available in RDP (release 11 update 5) were included in the analysis. Sequences were trimmed to the V1-V2 region and all sequences

of poor quality (≥1 N per sequence) and those not completely covering the amplified region were deleted. Sequences appearing only once were deleted and only representative sequences are shown. GenBank accession numbers are given after and the RDP ID before the strain name. The amount of isolates having an identical sequence is given after the accession number. Sequences of type strains are marked by a red dot and phylotype sequences by a black dot. Source data are provided in Supplementary Data 1.

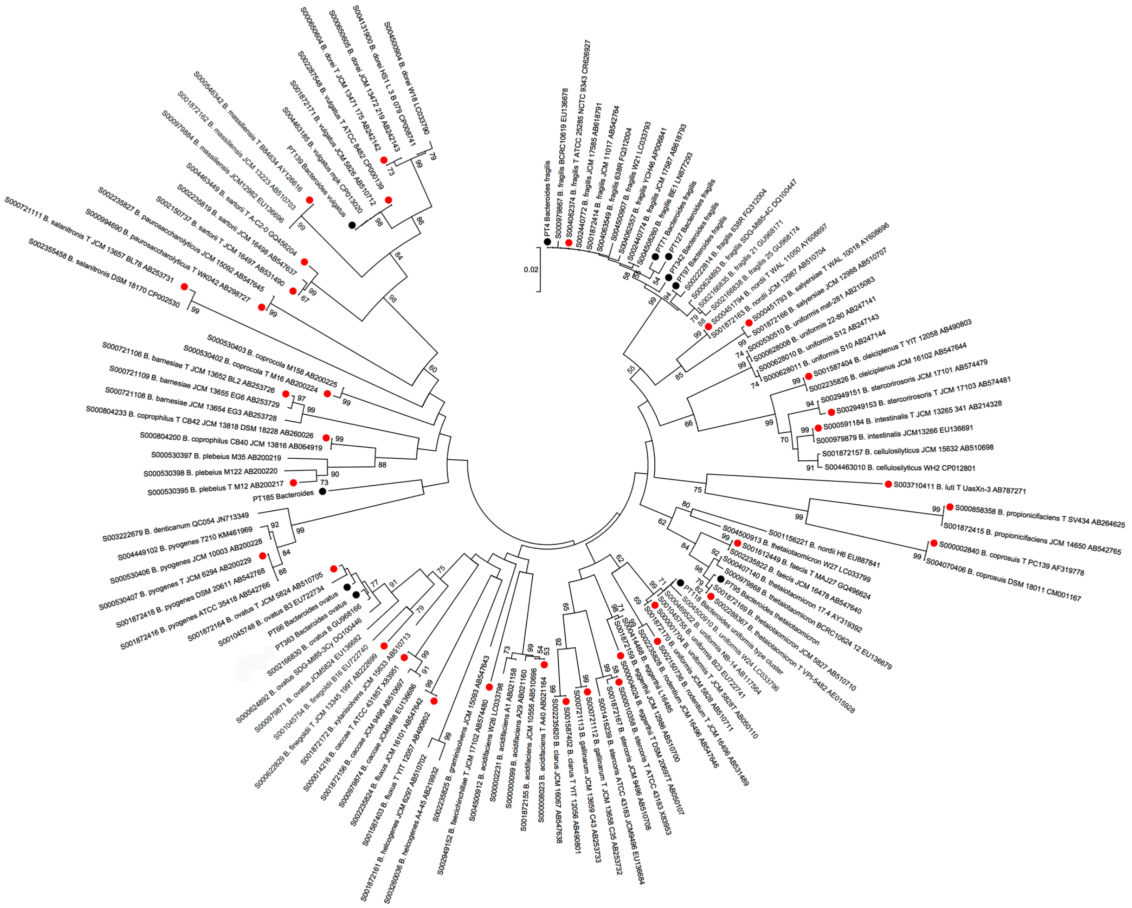

Supplementary Fig. 3: Taxonomic distribution of 16S rRNA gene sequences originating from *Bacteroides* spp. Sequences were aligned using MUSCLE and edited with SEAVIEW. The phylogenetic tree was constructed with MEGA7 using the neighbor-joining algorithm with p-distance correction and pairwise deletion of gaps and missing data. A total of 100 bootstrap replications were done to test for branch robustness (bootstrap values >50% are shown adjacent to each cluster node). Sequences of all of isolates with species level annotation available in RDP (release 11 update 5) were included in the analysis. Sequences were trimmed to the V1-V2 region and all sequences of poor quality (≥1 N per sequence) and those not completely covering the amplified region were deleted. Only representative sequences are shown. GenBank accession

numbers are given after and the RDP ID before the strain name. Sequences of type strains are marked by a red dot and phylotype sequences by a black dot. Source data are provided in Supplementary Data 1.

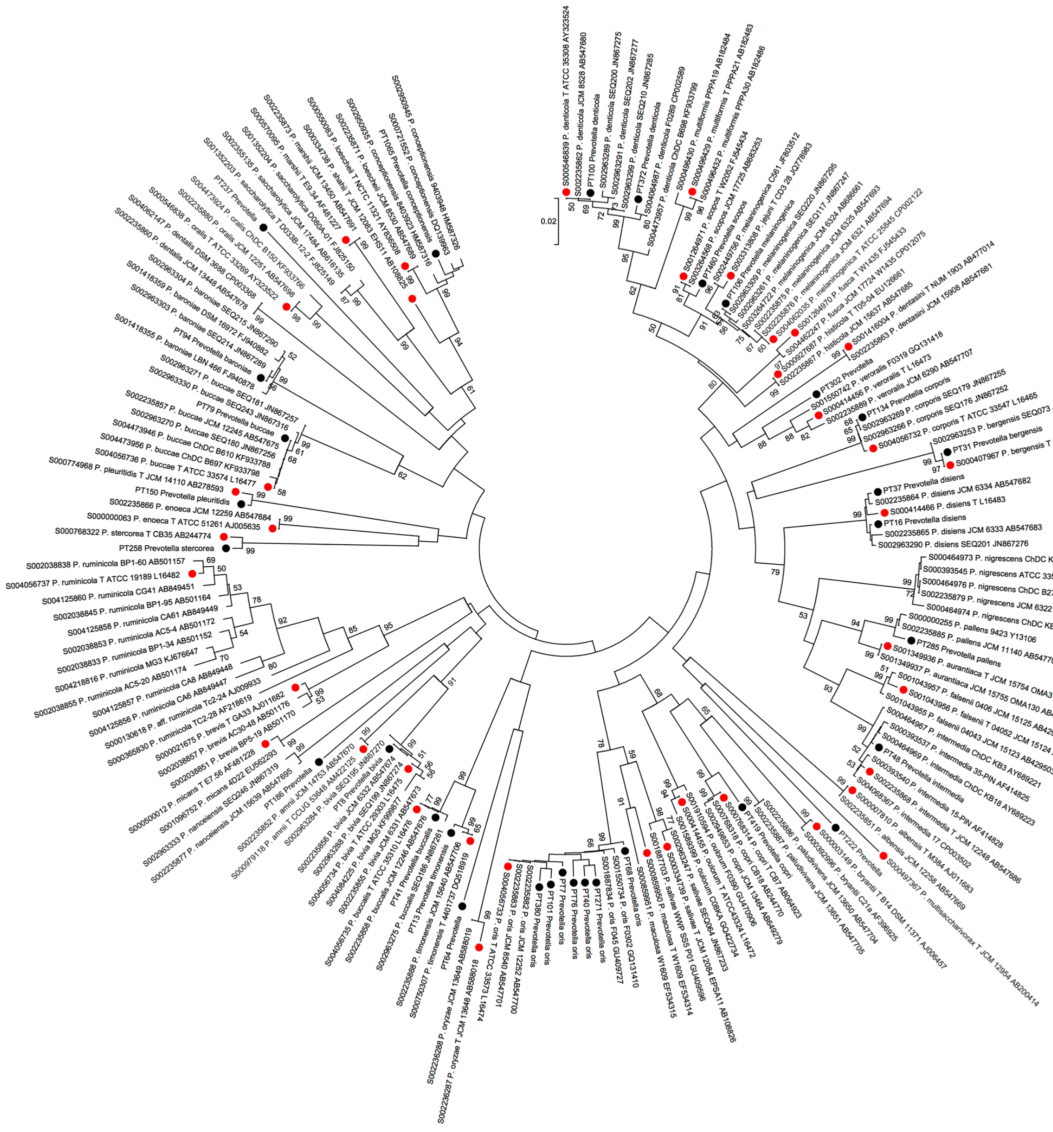

Supplementary Fig. 4: Taxonomic distribution of 16S rRNA gene sequences originating from *Prevotella* spp. Sequences were aligned using MUSCLE and edited with SEAVIEW. The phylogenetic tree was constructed with MEGA7 using the neighbor-joining algorithm with p-distance correction and pairwise deletion of gaps and missing data. A total of 100 bootstrap replications were done to test for branch robustness (bootstrap values >50% are shown adjacent to each cluster node). Sequences of all of

isolates with species level annotation available in RDP (release 11 update 5) were included in the analysis. Sequences were trimmed to the V1-V2 region and all sequences of poor quality (≥1 N per sequence) and those not completely covering the amplified region were deleted. Only representative sequences are shown. GenBank accession numbers are given after and the RDP ID before the strain name. Sequences of type strains are marked by a red dot and phylotype sequences by a black dot. Source data are provided in Supplementary Data 1.

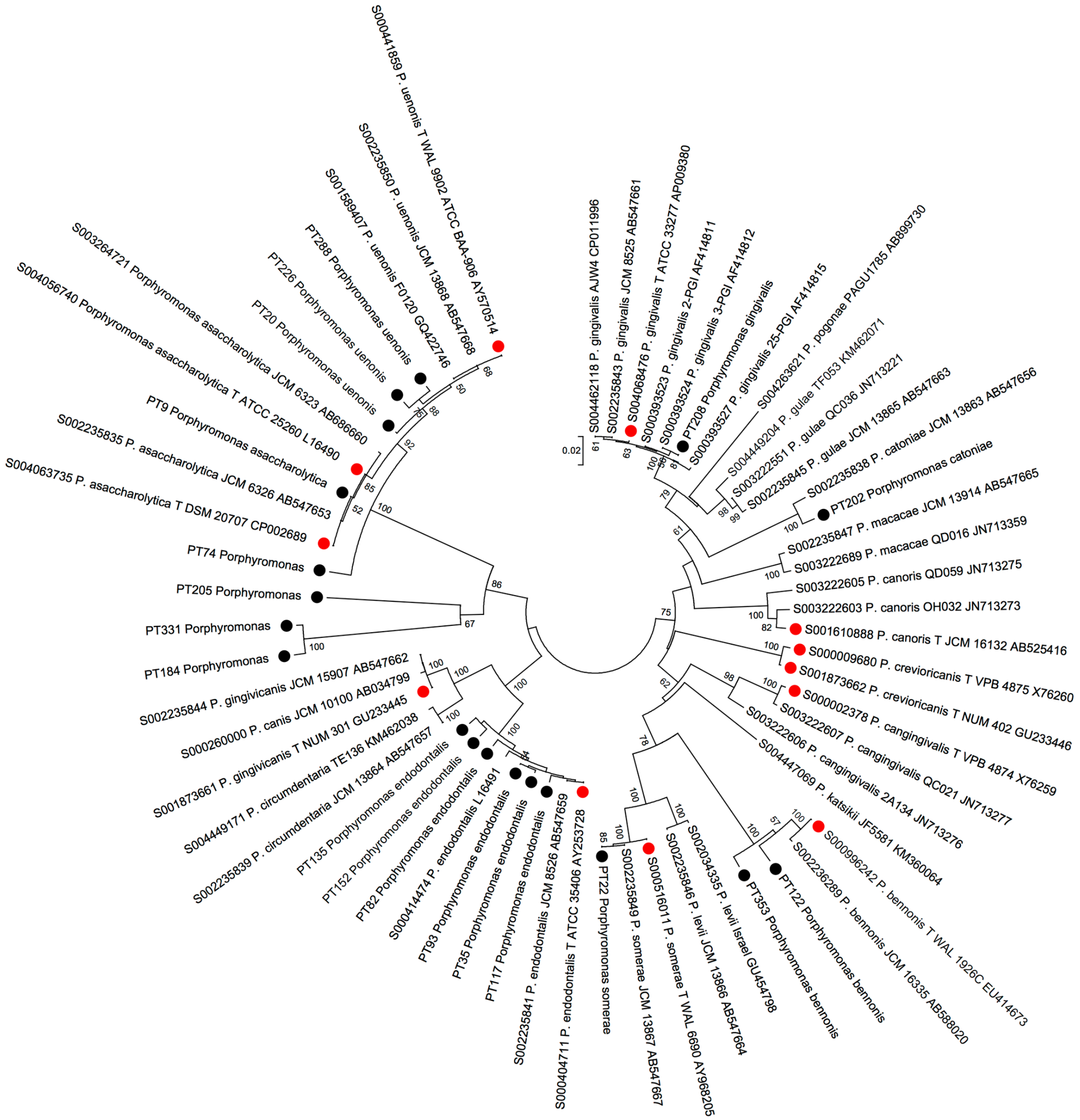

Supplementary Fig. 5: Taxonomic distribution of 16S rRNA gene sequences originating from *Porphyromonas* spp. Sequences were aligned using MUSCLE and edited with SEAVIEW. The phylogenetic tree was constructed with MEGA7 using the neighbor-joining algorithm with p-distance correction and pairwise deletion of gaps and missing data. A total of 100 bootstrap replications were done to test for branch robustness (bootstrap values >50% are shown adjacent to each cluster node). Sequences of all of

isolates with species level annotation available in RDP (release 11 update 5) were included in the analysis. Sequences were trimmed to the V1-V2 region and all sequences of poor quality (≥1 N per sequence) and those not completely covering the amplified region were deleted. Only representative sequences are shown. GenBank accession numbers are given after and the RDP ID before the strain name. Sequences of type strains are marked by a red dot and phylotype sequences by a black dot. Source data are provided in Supplementary Data 1.

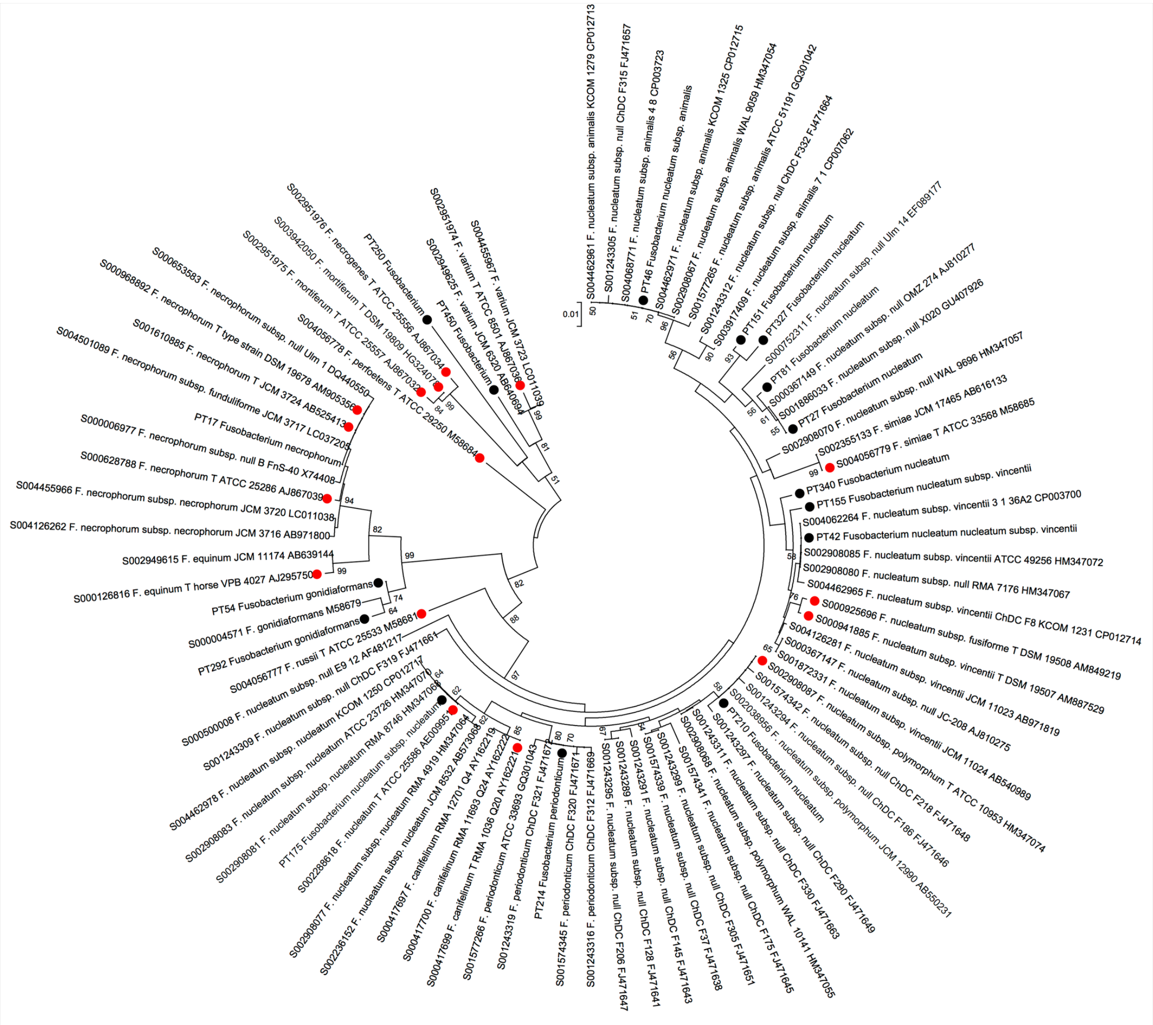

Supplementary Fig. 6: Taxonomic distribution of 16S rRNA gene sequences originating from *Fusobacterium* spp. Sequences were aligned using MUSCLE and edited with SEAVIEW. The phylogenetic tree was constructed with MEGA7 using the neighbor-joining algorithm with p-distance correction and pairwise deletion of gaps and missing data. A total of 100 bootstrap replications were done to test for branch robustness (bootstrap values >50% are shown adjacent to each cluster node). Sequences of all of isolates with species level annotation available in RDP (release 11 update 5) were included in the analysis. Sequences were trimmed to the V1-V2 region and all sequences of poor quality (≥1 N per sequence) and those not completely covering the amplified

region were deleted. Only representative sequences are shown. GenBank accession numbers are given after and the RDP ID before the strain name. Sequences of type strains are marked by a red dot and phylotype sequences by a black dot. Source data are provided in Supplementary Data 1.

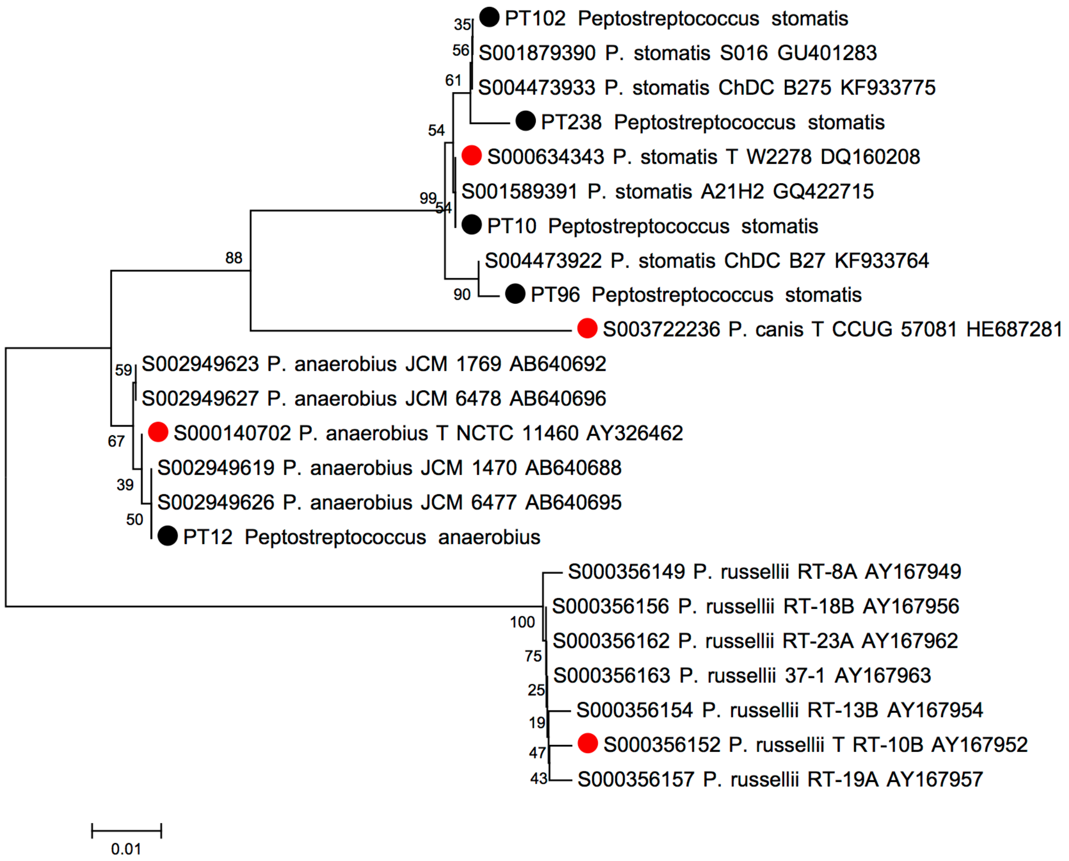

Supplementary Fig. 7: Taxonomic distribution of 16S rRNA gene sequences originating from *Peptostreptococcus* spp. Sequences were aligned using MUSCLE and edited with SEAVIEW. The phylogenetic tree was constructed with MEGA7 using the neighbor-joining algorithm with p-distance correction and pairwise deletion of gaps and missing data. A total of 100 bootstrap replications were done to test for branch robustness (bootstrap values >50% are shown adjacent to each cluster node). Sequences of all of isolates with species level annotation available in RDP (release 11 update 5) were included in the analysis. Sequences were trimmed to the V1-V2 region and all sequences of poor quality (≥1 N per sequence) and those not completely covering the amplified region were deleted. Only representative sequences are shown. GenBank accession numbers are given after and the RDP ID before the strain name. Sequences of type strains

are marked by a red dot and phylotype sequences by a black dot. Source data are provided in Supplementary Data 1.

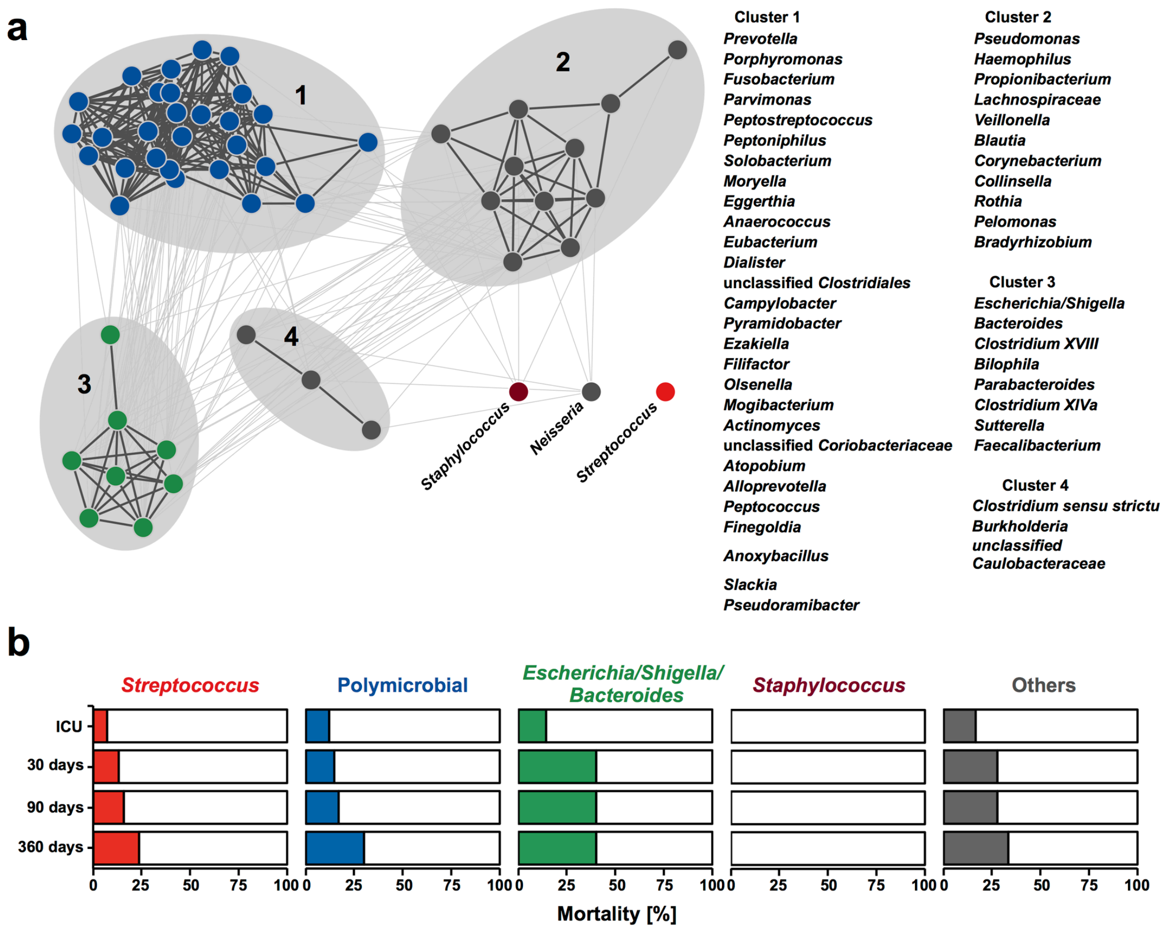

Supplementary Fig. 8: Distinct clusters of co-ocurring bacterial genera in NSTIs are associated with similar mortalities. a, Clusters were identified by divisive graph clustering of the co-occurrence network using the Markov cluster algorithm. Bacterial taxa associated with each identified network module are given on the right of the graph. **b**, Mortality associated with each identified type of NSTI (*Streptococcus* n = 69, polymicrobial n = 42, *Escherichia/Shigella/Bacteroides* n = 5, *Staphylococcus* n = 5, Others n = 18). Timepoints are given relative to the time of discharge from the ICU (days). Patients were grouped into pathotypes based on the microbial composition identified in the infected tissue (see Figure 2). Source data are provided in Supplementary Data 3, 4 and Table 1.

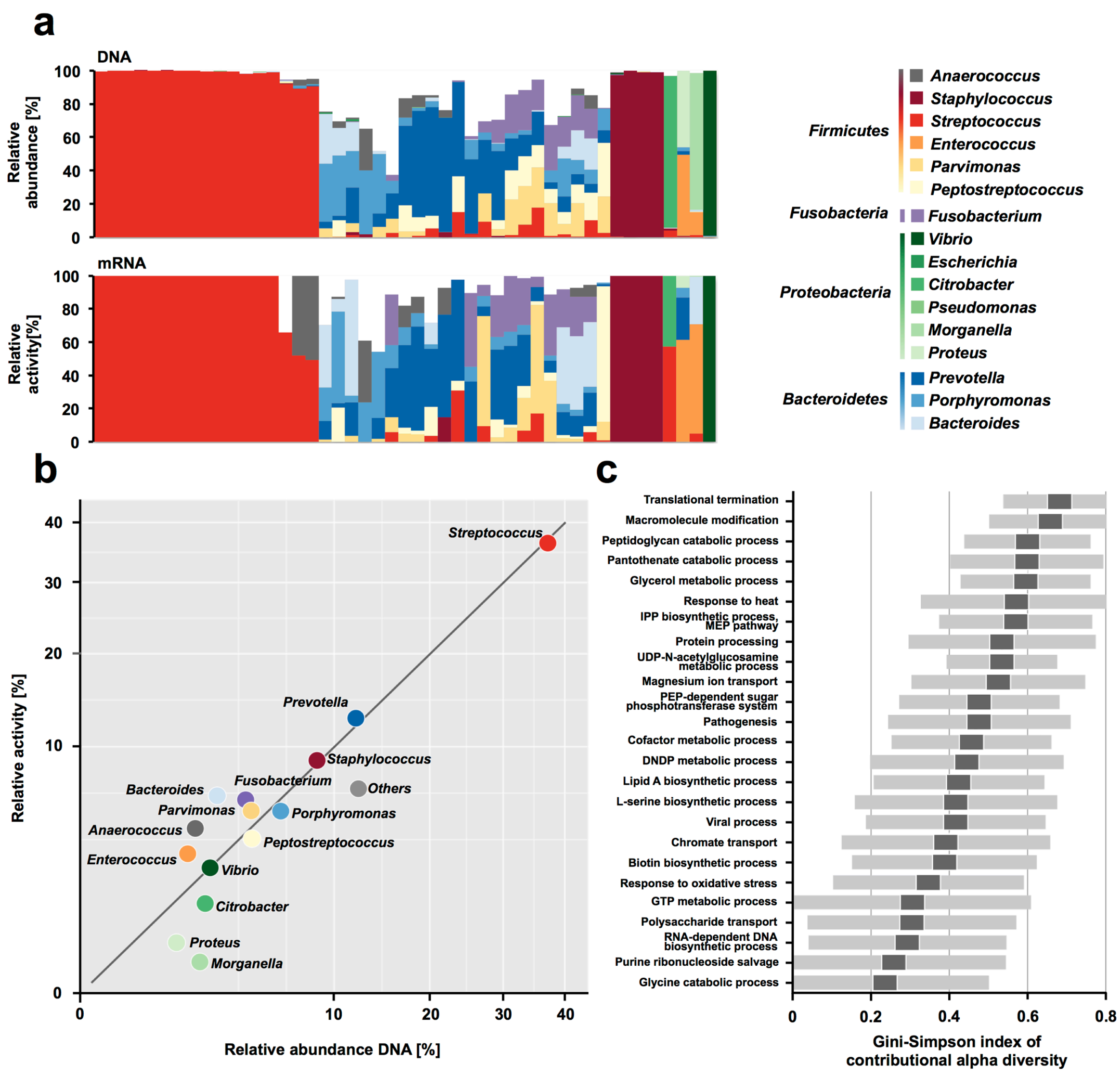

Supplementary Fig. 9: Bacterial transcriptional activity mirrors their relative abundance as determined by 16S rRNA sequencing. a, Relative abundance (top) and relative activity (bottom) of the highly abundant bacterial genera. Relative activity is defined as the relative contribution of a genus to the total transcriptional activity of the bacterial community. **b**, Average genus transcriptional activity correlated with the relative genus level abundance over all investigated tissue specimens (*n*=47). Pearson's *r*=0.972, *t*=15.045, *p*-value = 1.331e^{-09}. **c**, Bacterial alpha-diversity of the genus-level transcriptional contribution to the 25 GO-terms with the highest associated gene

expression in polymicrobial NSTIs. Dark center bar, mean; grey error bars, s.d. Source data are provided in Supplementary Data 2, 5 and 10.

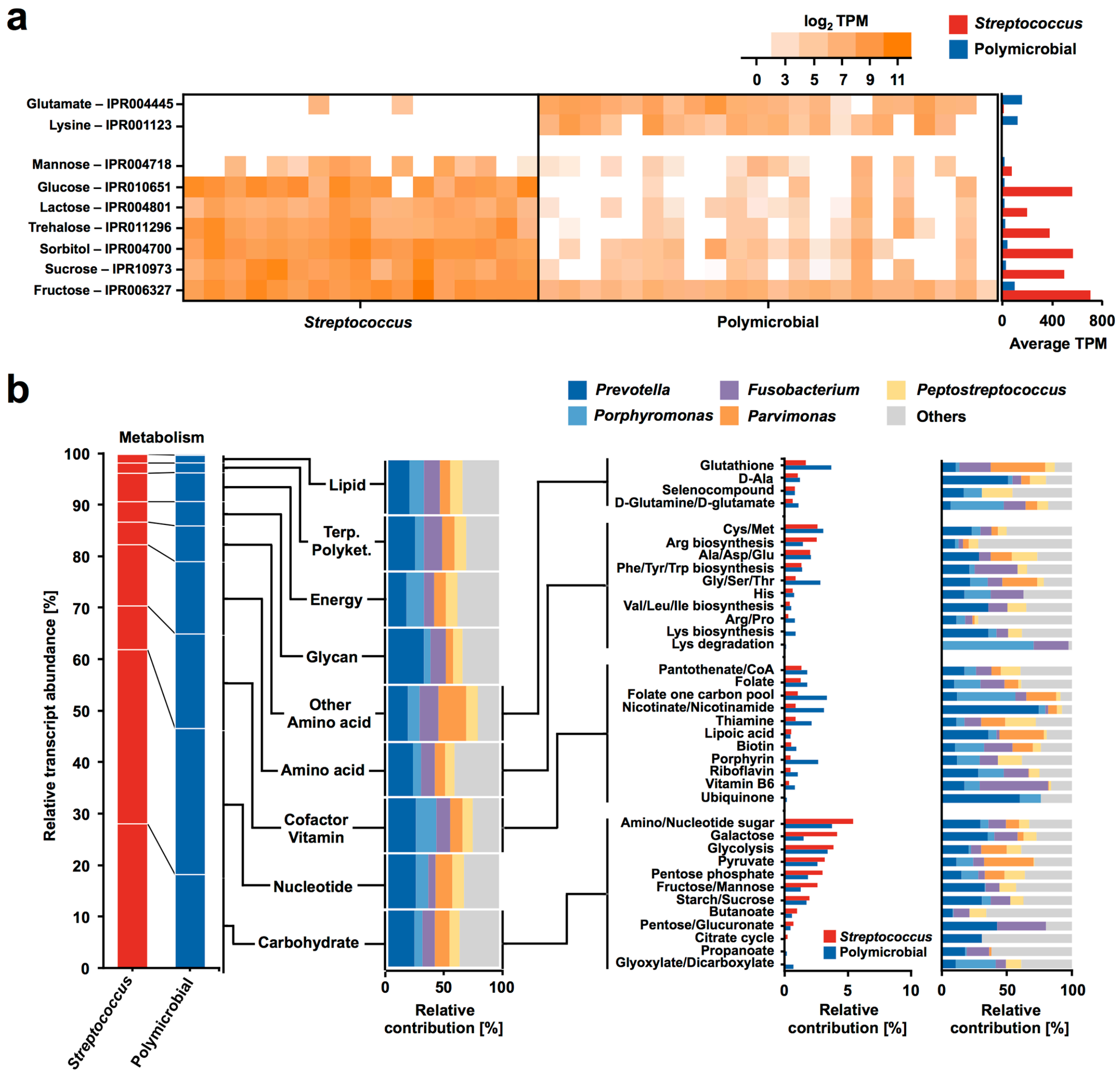

Supplementary Fig. 10: *Streptococcus* spp. and polymicrobial communities utilized different metabolic pathways during NSTIs. a, Summed expression of genes ($\log_2$ TPM) coding for transporter IPR domains associated with the GO terms ‘amino acid transport’ and ‘carbohydrate transport’ by bacterial communities in individual patients. Bars depict the average expression for all polymicrobial (*n*=22) respectively streptococcal (*n*=17) NSTIs. **b**, Metabolic profiles as characterized using the KEGG onthology of *Streptococcus* spp. respectively the polymicrobial communities during NSTIs. Average activity (left) and relative contribution of selected genera (mid left) to KEGG metabolic

pathway categories. Relative contribution of specific pathways to the total metabolic activity characterized by KEGG onthology (mid right) and relative contribution of selected genera to each pathway (right). Source data are provided in Supplementary Data 10 or as source data file.

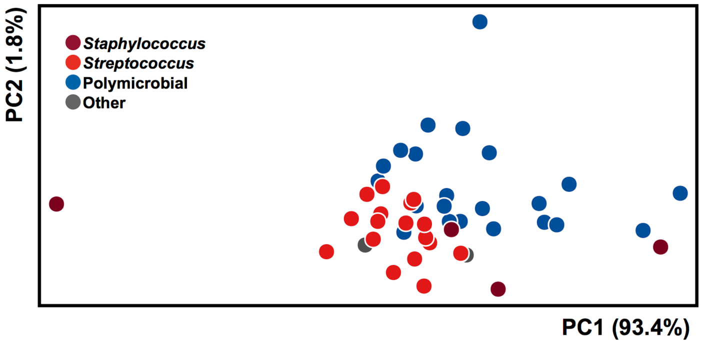

Supplementary Fig. 11: Human tissue activities differ significantly between polymicrobial and streptococcal NSTIs. PCA of the global transcriptional profile of infected tissue indicates differences between polymicrobial and streptococcal NSTIs. Source data are provided in Supplementary Data 7.

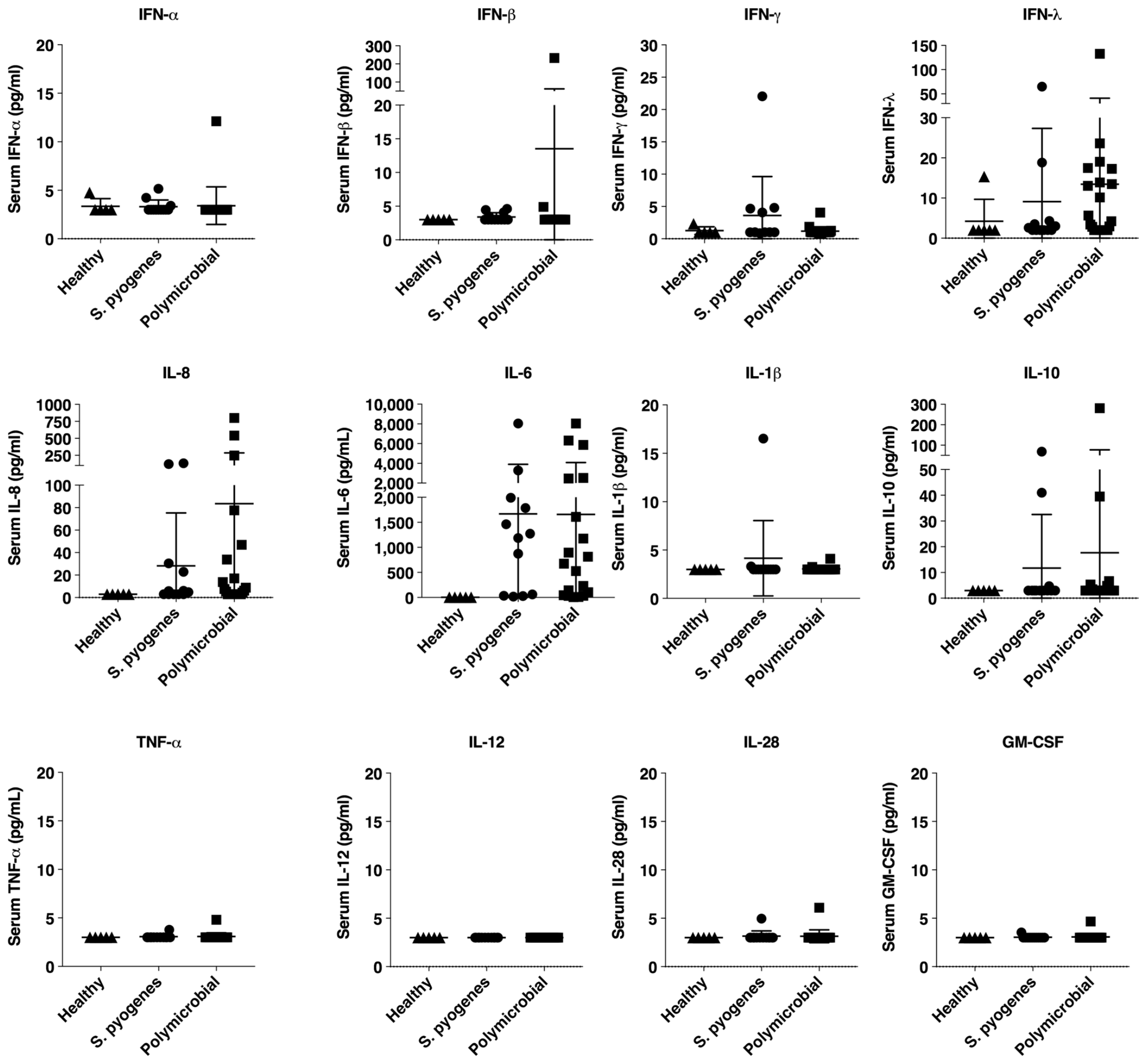

Supplementary Fig. 12: Plasma levels of interferon-inducible mediators that do not differ in a NSTI patient training cohort. The levels of 12 interferon-inducible mediators were measured in the plasma of patients with *S. pyogenes* (n=12) or polymicrobial (n=22) NSTIs, or healthy controls (n=5) by a multiplex beads array. The mean value (±s.d.) is indicated by a horizontal line. Source data are available as a source data file.

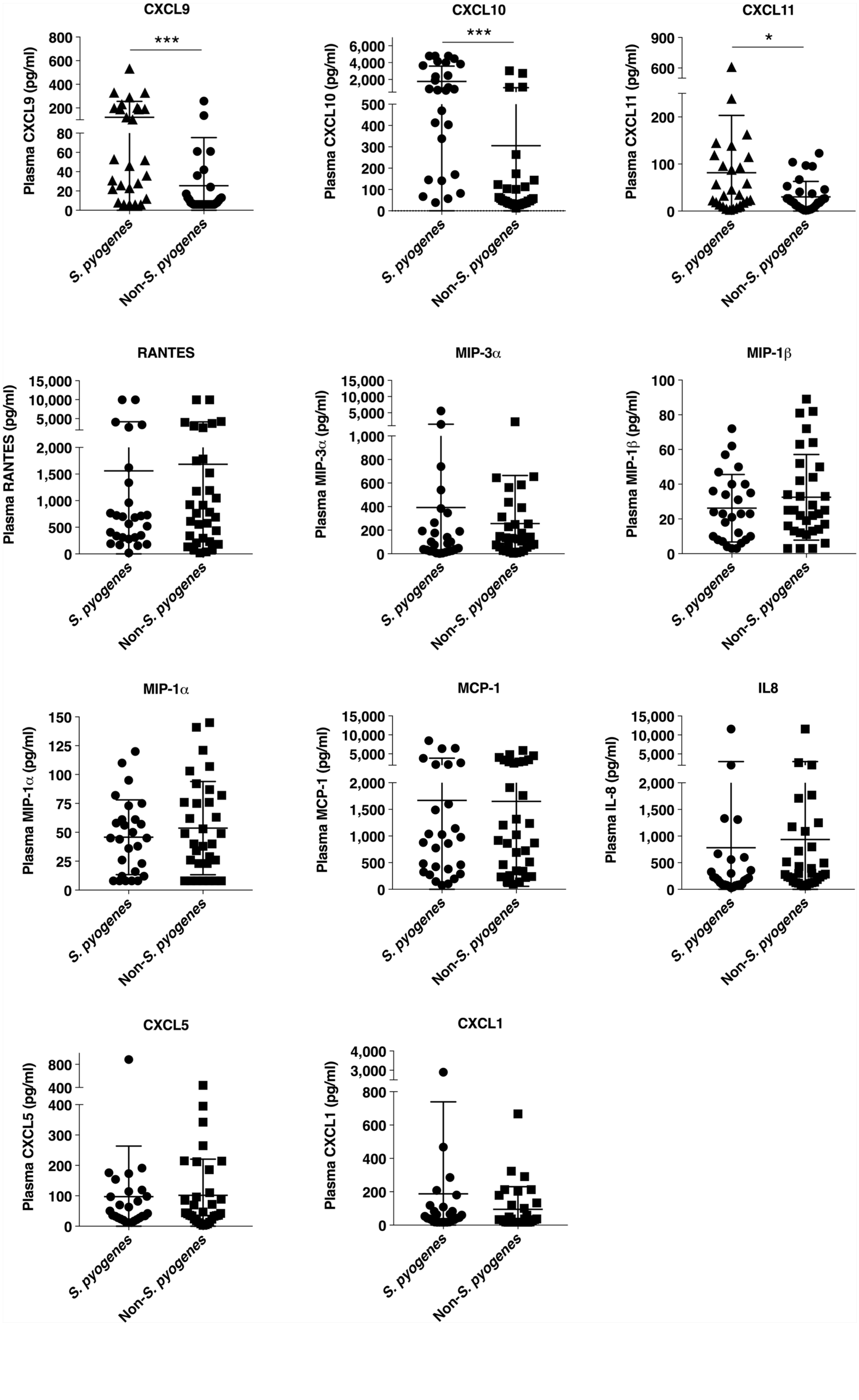
CXCL9
***
Plasma CXCL9 (pg/ml)
0 20 40 60 80 200 400 600 800
CXCL10
***
Plasma CXCL10 (pg/ml)
0 100 200 300 400 500 2,000 4,000 6,000
CXCL11
*
Plasma CXCL11 (pg/ml)
0 100 200 600 900
RANTES
Plasma RANTES (pg/ml)
0 500 1,000 1,500 2,000 5,000 10,000 15,000
MIP-3α
Plasma MIP-3α (pg/ml)
0 200 400 600 800 1,000 5,000 10,000 15,000
MIP-1β
Plasma MIP-1β (pg/ml)
0 20 40 60 80 100
MIP-1α
Plasma MIP-1α (pg/ml)
0 25 50 75 100 125 150
MCP-1
Plasma MCP-1 (pg/ml)
0 500 1,000 1,500 2,000 5,000 10,000 15,000
IL8
Plasma IL-8 (pg/ml)
0 500 1,000 1,500 2,000 5,000 10,000 15,000
CXCL5
Plasma CXCL5 (pg/ml)
0 100 200 300 400 400 800
CXCL1
Plasma CXCL1 (pg/ml)
0 200 400 600 800 3,000 4,000
S. pyogenes
Non-S. pyogenes

Supplementary Fig. 13: Plasma levels of several chemokines in an independent cohort of NSTIs patients. The chemokines levels were measured in the plasma of patients with NSTIs either caused by *S. pyogenes* (n=27) or by other microbial etiologies (n=32). The mean value (±s.d.) is indicated by a horizontal line. Statistical significance was evaluated using ordinary one way ANOVA with * p-value < 0.05; *** p-value < 0.001. Source data are available as a source data file.

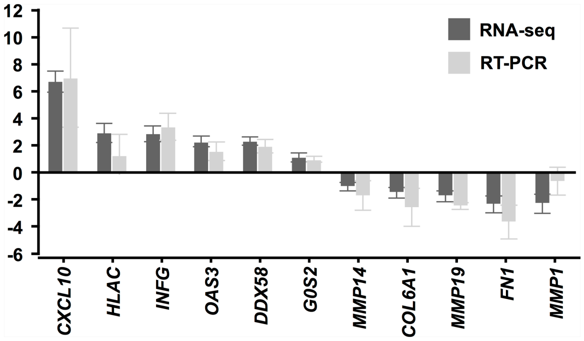

Supplementary Fig. 14: Validation of the fold-changes of selected genes between the infected tissue of patients with polymicrobial respectively streptococcal NSTIs as determined via RNA-seq data using qRT-PCR. Error bars, standard error. Source data are available as a source data file.

Supplementary Tables

Supplementary Table 1: Permutational Multivariate Analysis of Variance (PERMANOVA): pairwise comparisons between sampled sites.

	t-value	p-value (perm)	Unique perms	p-value (MC)	Degrees of freedom
U/L	1.045	0.333	997	0.328	53
U/H	2.754	0.001	998	0.001	30
U/A	2.351	0.001	999	0.001	39
U/T	1.253	0.154	998	0.176	28
L/H	3.045	0.001	999	0.001	49
L/A	2.680	0.001	998	0.001	58
L/T	1.093	0.255	999	0.281	47
H/A	1.414	0.032	995	0.050	35
H/T	2.014	0.007	998	0.007	24
A/T	1.411	0.053	996	0.053	33

The resemblance matrix was generated using the Bray-Curtis algorithm. PERMANOVA was performed using type III (partial) sums of squares with a fixed effects sum to zero for mixed terms. Exact *p*-values (*p*-value perm) were generated using unrestricted permutation of the raw data (number of permutations 999). Monte Carlo simulations were also performed and *p*-values computed (*p*-value MC). Differences between groups were considered significant if *p* (perm and MC) were <0.05. The sampled sites compated were U, upper extremities; L, lower extremities; H, head/neck; A, anogenital region; T, thorax/abdomen.

Supplementary Table 2: Differences in global tissue expression profiles during NSTIs**.**

	t-value	*p-value* (perm)	Unique perms	*p*-value (MC)	Degrees of freedom
Streptococcus, Polymicrobial	3.7838	0.001	999	0.02	37
Streptococcus, Staphylococcus	0.42079	0.551	924	0.717	19
Streptococcus, Others	1.8915	0.054	926	0.043	19
Polymicrobial, Staphylococcus	1.0235	0.268	970	0.324	24
Polymicrobial, Others	1.1208	0.272	958	0.274	24
Staphylococcus, Others	0.21331	0.913	35	0.89	6

Tissue expression profiles were compared using the permutational multivariate analysis of variance (PERMANOVA). The resemblance matrix was generated based on euclidean distance measurements using type III (partial) sums of squares with a fixed effects sum to zero for mixed terms. Exact *p*-values (*p*-value perm) were generated using unrestricted permutation of the raw data (number of permutations 999). *P*-values were reported for each pair of conditions. Monte Carlo simulations were performed and *p*-values computed (*p*-value MC) as low unique permutations were obtained for some pairwise comparisons. Differences between groups were considered significant if *p* (perm and MC) were <0.05.

Supplementary Table 3. Primer sequences used for qRT-PCR

target gene	forward primer	reverse primer
COL6A1	AGGGACAGGTACTACCGCTG	GTGTAGGTGCCCTTCCCAAA
CXCL10	TCCACGTGTTGAGATCATTGCTA	TGCATCGATTTTGCTCCCCT
DDX58	AAAGCTAGTGAGGCACAGCC	GCACCTCTTCCTCCCTAAACC
FN1	GAACAAACACTAATGTTAATTGCCC	CGGGAATCTTCTCTGTCAGCC
G0S2	CACTAAGGTCATTCCCGCCT	AGCACGTACAGCTTCACCAT
HLAC	GGTTGTCCTAGCTGTCCTTGG	GCTGTCTCAGGCTTTACAAGTG
INFG	GGCTTTTCAGCTCTGCATCG	TCTGTCACTCTCCTCTTTCCA
MMP1	TCTGGAAGGGCAAGGACTCT	TCAACTTGCCTTTGTCTTCTTTCT
MMP14	TCCAGCAACTTTATGGGGGT	TTCCCGTCACAGATGTTGGG
MMP19	TGTTCCTCTTTAAGGGCTCCG	GTTGAGGCGCCAGTAGACTT
OAS3	GGCTGGAACTCCTGACTGTG	GATGATAGGCCTGGGCTTCTG
