# Supplementary material for: Molecular profiling of tissue biopsies reveals unique signatures associated with streptococcal necrotizing soft tissue infections
Source: Nat Commun. 2019 Aug 26;10:3846. doi: 10.1038/s41467-019-11722-8 (PMC6710258; doi:10.1038/s41467-019-11722-8)
Supplement: Supplementary file 3 — Description of Additional Supplementary Files [file 41467_2019_11722_MOESM3_ESM.pdf]

Description of Additional Supplementary Files

File Name: Supplementary Data 1
Description: Nucleotide sequences of all phylotypes determined using Illumina-based amplicon deep-sequencing, their phylogenetic annotation and relative abundance across all 148 tissue biopsies.

File Name: Supplementary Data 2
Description: Sequence count data of genera after libraries have been rarefied to the lowest sequence count of 3176 sequences per sample.

File Name: Supplementary Data 3
Description: Sample classification. The sample number (as ordered in Figure 1a), sample ID, location of the infection (if present at multiple locations no location is given), and the Gini-Simpson index (based on phylotype data or genus data) are given. It is also indicted if RNASeq data are available. Moreover, classification based on average linkage hierarchical agglomerative clustering and cluster identification using the J-index is given. In case of cluster 1 samples, the most abundant *Streptococcus* species is given. Samples used in the training cohort (T) and the external cohort (E) to discriminate between streptococcal and polymicrobial NSTIs are also indicated.

File Name: Supplementary Data 4
Description: Co-occurence model node and edge description.

File Name: Supplementary Data 5
Description: Gene onthology analysis.

File Name: Supplementary Data 6
Description: Virulence factor InterPro domain database.

File Name: Supplementary Data 7
Description: RNAseq Count Table generated by pseudoalignment against the human reference assembly GRCh38.p10 using kallisto.

File Name: Supplementary Data 8
Description: Differential gene expression analysis of human tissue transcription between polymicrobial and streptococcal NSTIs using DESeq2.

File Name: Supplementary Data 9
Description: RefSeq genome assemblies used for genus-specific metatranscriptomes.

File Name: Supplementary Data 10
Description: Normalized count table generated by pseudoalignment against sample-specific reference databases using kallisto.
