# Supplementary material for: Molecular profiling of tissue biopsies reveals unique signatures associated with streptococcal necrotizing soft tissue infections
Source: Nat Commun. 2019 Aug 26;10:3846. doi: 10.1038/s41467-019-11722-8 (PMC6710258; doi:10.1038/s41467-019-11722-8)
Supplement: Supplementary file 14 — Reporting Summary [file 41467_2019_11722_MOESM14_ESM.pdf]

nature research

Corresponding author(s): Dietmar H. Pieper

Reporting Summary

Nature Research wishes to improve the reproducibility of the work that we publish. This form provides structure for consistency and transparency in reporting. For further information on Nature Research policies, see Authors & Referees and the Editorial Policy Checklist.

Statistical parameters

When statistical analyses are reported, confirm that the following items are present in the relevant location (e.g. figure legend, table legend, main text, or Methods section).

n/a	Confirmed	
☐	☒	The exact sample size (*n*) for each experimental group/condition, given as a discrete number and unit of measurement
☒	☐	An indication of whether measurements were taken from distinct samples or whether the same sample was measured repeatedly
☐	☒	The statistical test(s) used AND whether they are one- or two-sided<br>*Only common tests should be described solely by name; describe more complex techniques in the Methods section.*
☐	☒	A description of all covariates tested
☐	☒	A description of any assumptions or corrections, such as tests of normality and adjustment for multiple comparisons
☐	☒	A full description of the statistics including central tendency (e.g. means) or other basic estimates (e.g. regression coefficient) AND variation (e.g. standard deviation) or associated estimates of uncertainty (e.g. confidence intervals)
☐	☒	For null hypothesis testing, the test statistic (e.g. *F*, *t*, *r*) with confidence intervals, effect sizes, degrees of freedom and *P* value noted<br>*Give P values as exact values whenever suitable.*
☒	☐	For Bayesian analysis, information on the choice of priors and Markov chain Monte Carlo settings
☐	☒	For hierarchical and complex designs, identification of the appropriate level for tests and full reporting of outcomes
☐	☒	Estimates of effect sizes (e.g. Cohen's *d*, Pearson's *r*), indicating how they were calculated
☐	☒	Clearly defined error bars<br>*State explicitly what error bars represent (e.g. SD, SE, CI)*

Our web collection on statistics for biologists may be useful.

Software and code

Policy information about availability of computer code

Data collection	Reference assemblies for dual RNA-seq analysis were downloaded from the NCBI RefSeq, respectively Ensembl database (see Method section). Reference 16S isolate sequences for species level annotation were downloaded from the Ribosomal Database Project (RDP, release 11 update 5).
Data analysis	For 16S rRNA based taxonomic characterization raw read were merged using the RDP assemlber, aligned using MOTHUR, and analyzed against the RDP database using the Seqmatch function (see Method section for details).<br>Raw RNA-seq reads were trimmed using fastq-mcf and pseudoaligned to the respective reference assemblies using kallisto. Differential gene expression analysis of the human transcriptional profiles was performed using the DESeq2 in R and enrichment analysis was conducted using DAVID Bioinformatic resources. Data were visualized and analyzed using Cytoscape (packages Enrichment Map, CytoCluster). Bacterial expression profiles were further annotated using InterProScan 5 using the KEGG, GO, PFAM and InterPro databases. Genus specific gene clusters were constructed using BLAST following the UniRef50 parameters to identify homologous genes (see Methods). Virulence factors were further annotated using the Virulence Factor Database (VFDB, December 2017). A detailed description is provided in the method section.

For manuscripts utilizing custom algorithms or software that are central to the research but not yet described in published literature, software must be made available to editors/reviewers upon request. We strongly encourage code deposition in a community repository (e.g. GitHub). See the Nature Research guidelines for submitting code & software for further information.

Data

Policy information about availability of data

All manuscripts must include a data availability statement. This statement should provide the following information, where applicable:

- Accession codes, unique identifiers, or web links for publicly available datasets
- A list of figures that have associated raw data
- A description of any restrictions on data availability

The sequencing data generated during this study have been deposited in the Sequence Read Archive under BioProject accession PRJNA479582. The complete raw data underlying the manuscripts figures have been provided as Supplementary Tables and relevant associations of Supplementary Tables and Figures have been highlighted at the respective position in the manuscript.

Field-specific reporting

Please select the best fit for your research. If you are not sure, read the appropriate sections before making your selection.

☒ Life sciences ☐ Behavioural & social sciences ☐ Ecological, evolutionary & environmental sciences

For a reference copy of the document with all sections, see nature.com/authors/policies/ReportingSummary-flat.pdf

Life sciences study design

All studies must disclose on these points even when the disclosure is negative.

Sample size	While sample availability in this non-interventional, observational study that leverages existing specimens for which recruitment, enrollment, and sample collection had concluded, was pre-determined, we set sample sizes for comparative transcriptional profiling of polymicrobial and streptococcal NSTIs (dual RNA-seq) so that our sample size would have >80% power to reject the null hypothesis at a significance level of 0.05 for a fold change of 2.
Data exclusions	Sequencing data was excluded if specimens showed insufficient input material or quality (RNA integrity) after repeated DNA/ RNA extraction or insufficient depth (RNAseq <5 million total reads, 16S sequencing <3000 total reads) following sequencing.
Replication	As the studies findings result from observational data from a unique patient cohort biological replication was not applicable. We performed selective technical replication of 16S rRNA sequencing results which indicated high reproducibility between technical replicates. All code for for the taxonomic and functional analysis and annotation has been been reported in previous publications (see Methods section for citations).
Randomization	Patients with clinically diagnosed necrotizing soft tissue infection were included in this study. Patients were grouped into different groups according to the identified microbial composition in the infected tissue as determined via 16S rRNA sequencing using divisive and hierarchical clustering as described in the Method section.
Blinding	Investigators were not blinded during data collection or analysis.

Reporting for specific materials, systems and methods

Materials & experimental systems

n/a	Involved in the study	
☒	☐	Unique biological materials
☒	☐	Antibodies
☒	☐	Eukaryotic cell lines
☒	☐	Palaeontology
☒	☐	Animals and other organisms
☐	☒	Human research participants

Methods

n/a	Involved in the study	
☒	☐	ChIP-seq
☒	☐	Flow cytometry
☒	☐	MRI-based neuroimaging

Human research participants

Policy information about studies involving human research participants

Population characteristics	The studies cohort is a randomly selected subset of the INFECT study cohort (ClinicalTrials.gov, NCT01790698). Our subset of this cohort consists of 148 patients with clinically diagnosed necrotizing soft tissue infections (88 male, 60 female) with a median age of 60 years (range 20 to 90 years). Patients were recruited in 5 hospitals across Scandinavia (in our subset: 82 Rigshospitalet

Copenhagen, 23 Karolinska University Hospital, 4 Blekingesjukhuset Karlskrona, 17 Sahlgrenska University Hospital, 22 University of Bergen). Analyzed tissue biopsies and blood samples were taken on the day of admission to the participating hospital.

Recruitment	Patients were recruited in the framework of the EU-funded INFECT project (www.fp7infect.eu) Written informed consent was obtained from all patients or their surrogate. The INFECT study is registered at ClinicalTrials.gov (NCT01790698) and was conducted in accordance with the Declaration of Helsinki and with approval of all relevant authorities.
